# Tool-use training in augmented reality: plasticity of forearm body schema does not predict sense of ownership or agency in older adults

**DOI:** 10.1007/s00221-023-06645-2

**Published:** 2023-06-12

**Authors:** Amir Jahanian Najafabadi, Dennis Küster, Felix Putze, Ben Godde

**Affiliations:** 1 Department of Cognitive Neuroscience, Bielefeld University, 33501 Bielefeld, Germany; 2 School of Business, Social and Decision Sciences, Constructor University Bremen, 28759 Bremen, Germany; 3 Department of Computer Science, University of Bremen, 28359 Bremen, Germany

**Keywords:** Virtual tool-use, Forearm body schema, Sense of ownership and agency, Somatosensory representation, Older adults

## Abstract

In young adults (YA) who practised controlling a virtual tool in augmented reality (AR), the emergence of a sense of body ownership over the tool was associated with the integration of the virtual tool into the body schema (BS). Agency emerged independent of BS plasticity. Here we aimed to replicate these findings in older adults (OA). Although they are still able to learn new motor tasks, brain plasticity and learning capacity are reduced in OA. We predicted that OA would be able to gain control over the virtual tool indicated by the emergence of agency but would show less BS plasticity as compared to YA. Still, an association between BS plasticity and body ownership was expected. OA were trained in AR to control a virtual gripper to enclose and touch a virtual object. In the visuo-tactile (VT) but not the vision-only (V) condition, vibro-tactile feedback was applied through a CyberTouch II glove when the tool touched the object. BS plasticity was assessed with a tactile distance judgement task where participants judged distances between two tactile stimuli applied to their right forearm. Participants further rated their perceived ownership and agency after training. As expected, agency emerged during the use of the tool. However, results did not indicate any changes in the BS of the forearm after virtual tool-use training. Also, an association between BS plasticity and the emergence of body ownership could not be confirmed for OA. Similar to YA, the practice effect was stronger in the visuo-tactile feedback condition compared with the vision-only condition. We conclude that a sense of agency may strongly relate to improvement in tool-use in OA independent of alterations in the BS, while ownership did not emerge due to a lack of BS plasticity.

**Supplementary Information:**

The online version contains supplementary material available at 10.1007/s00221-023-06645-2.

## Introduction

One of the hallmark skills of humans is the ability to handle an object (the “tool”) to reach, manipulate or grasp while interacting with objects in the environment (Miller et al. 2017; Cardinali et al. 2012). According to Nabeshima et al. (2006), tool-use allows us to overcome the limitations of the bodies in our daily life, moving beyond the limits imposed by the lengths of one’s limbs or the type of one’s end-effector. Herewith, tool-use is particularly important for older adults (OA) who suffer from various sensorimotor and perceptual decline but are dependent on using tools in their daily life to maintain their independent living.

According to the dyadic model (for review: Cardinali et al. 2011; Head and Holmes 1911), the following two distinct subcomponents constitute the body representation: body image (BI) and body schema (BS; de Vignemont 2010; Dijkerman and de Haan 2007). The BI is a perceptual conscious representation of the body (Cardinali et al. 2012) and is involved in body perception, body affect (Cardinali et al. 2012; de Vignemont 2010; Gallagher 2005), and body concept (Segura-Valverde et al. 2017). The BS is seen as an unconscious sensorimotor representation of the body that is used for action planning and execution of movements (Martel et al. 2016; d’Angelo et al. 2018). In contrast to the BI which is seen as a stable representation of body shape and size (Cardinali et al. 2009), the BS has been defined as a more short-term representation (Cardinali et al. 2012). It flexibly updates with every change in the state of the body, e.g., due to growth and body lengthening accompanying maturation—or as an effect of tool-use (Cardinali et al. 2012, 2009). In this line, extensive experimental efforts in healthy young adults (YA) during the past two decades contributed to an understanding of how short- and long-term tool-use experience modifies the body image (BI) and body schema (BS) (Martel et al. 2019; Day et al. 2017; McCormack et al. 2011; Miller et al. 2014) and how a sense of body ownership and a sense of agency, and thus feelings of control over the tool and its movements emerge (Nava et al. 2018; Jung and Hughes 2016). However, less is known about effects of tool-use training on ownership and agency in OA.

While ownership over a tool refers to tools being perceived as belonging to one’s own body and the sense that “I am the one that is going to experience, e.g., when one’s body is moving regardless of whether voluntarily or involuntarily” (Gallagher 2000, p15), agency is the feeling that actions or events of a tool are produced by one’s own body, and that the agent is the cause of the tool’s action (Gallagher 2000, 2018).

Synchronous multisensory inputs from different modalities play a fundamental role in producing body ownership (Maravita et al. 2003). As described by Sposito et al. (2012), ownership, i.e., tools being perceived as belonging to one’s own body, is highly plastic, and involves multiple body representations in the human brain. Cardinali et al (2012) and de Vignemont (2007) proposed that the BS becomes a source of ownership because it constitutes the spatial content of the bodily sensations that localise bodily properties within the BS. Empirical studies support the notion of tool use-induced representational plasticity whereby the tool is integrated into the existent BS in the somatosensory cortex, resulting in a changed BI (Ma and Hommel 2015; Miller et al. 2014). Our own recent results suggest that the emergence of ownership may strongly relate to changes in the sensorimotor BS which then are likely to be reflected in an altered BI (Jahanian Najafabadi et al. 2022). Therefore, mostly bottom-up afferent information received from multisensory modalities contributes to the emergence of ownership (Ma and Hommel 2015; Tsakiris and Haggard 2005).

Agency is strongly dependent on efferent components, as actions are centrally generated. Further, a recent study found that agency plays a crucial role in mediating e.g., the effect of synchronous training and the possibility to sense the control over an external object, here a virtual hand, without perceiving it as part of the body (D’Angelo et al. 2018). These authors further suggested that a sense of agency plays a major role in the construction of the BS and peripersonal space (PPS) representations.

Studies on tool-use training in OA with reduced BS plasticity could contribute to a better understanding of the dependencies of ownership and agency on BS plasticity. The progressive decline of cognitive and sensorimotor abilities with ageing could potentially affect malleability of body representation (BR) and particularly BS in OA (Sorrentino et al. 2021; Raimo et al. 2019). In this line, an earlier study argued that impaired tool-use in OA is caused not only by age-related decline in motor function but also cognitive, particularly semantic dysfunction (Lesourd et al. 2016). However, the mechanism of how age-related sensorimotor alterations contribute to long-term changes in the BS requires more evidence, particularly for implicit plasticity of BS and BI, ownership, and agency (Raimo et al. 2019).

With respect to tool-use learning and representational plasticity in OA, a few studies have been conducted. Some of studies focused on attentional and perceptual components in tool-use performance. For example, a study by Bloesch et al. (2013) confirmed a lack of spatial compression (the tool-use effect on distance estimation) in OA and implied a failure of the visuomotor system in transforming PPS to extra personal spatial representations during tool-use (Costello et al. 2015). In the same vein, a recent study reported that tool-use action is affected by spatial and other aspects of perception and indirectly influenced by attention (Witt 2021). It was concluded that the effect of tool-use on spatial perception confirms that tools are incorporated into the existing BS and embodied without specified age-related components. These findings are particularly interesting, especially given that age differences indicated by these studies may significantly affect how OA plan and guide actions in their PPS compared with YA (Bloesch et al. 2013).

Furher research focused on the visual, motor and tactile aspects of sensorimotor learning in OA compared with YA. Ghafouri and Lestienne (2000) suggested that OA have difficulty integrating information from different sensory modalities that are required to form a stable spatial representation of their own body. In this line, a study by Teixeira and Lima (2009) found that in OA sensorimotor learning is more dependent on visual signals compared to visuomotor signals. A further study by Devlin and Wilson (2010) reported a decline in visuo-spatial processing and the integrity of BS-related information with age that is required to update the location of the whole body through the mental transformation process (Devlin and Wilson 2010). Costello and colleagues (2015, 2017) additionally reported that for OA the integration of visual-tactile modalities during tool-use is particularly important since it is one of the major challenges in older age.

Marotta et al. (2018) examined age effects on ownership induced by the rubber hand illusion (RHI) paradigm (Botvinick and Cohen 1998). They revealed that both YA and OA experienced feelings of ownership and localization of touch over the rubber hand after synchronous stroking, followed by a full incorporation of the rubber hand into the BS. They emphasised a flexible representation of the body, together with multisensory integration of conflicting visual, tactile, and proprioceptive information, leading to a full incorporation of the rubber hand into the internal body model (Marotta et al. 2018).

Graham et al. (2014) used a modified version of the RHI to study mechanisms of self-perception. In paradigm, participants receive a live image of their hand from a monitor in front of them which is called projected RH and creates the illusory sensation of their hand with a precise timing of synchronous or asynchronous stroking. The authors found a decreased subjective feeling of ownership with increasing age and a greater proprioceptive drift as a change in the location of participants’ finger relative to the location they felt the finger (Graham et al. 2014). Another RHI study also found a decreased subjective feeling of ownership and agency with increasing age but no changes in proprioceptive drift (Kállai et al. 2017). These authors reported a decreased integration of visuo-tactile stimuli in OA during synchronous stimulation and less induced illusion in the visuo-tactile condition compared to YA. No age effects on multisensory integration were observed when visuo-proprioceptive feedback was synchronously presented during the task (Kállai et al. 2017).

Most prior approaches lacked effective means to separate the tactile and visual modalities such that tactile feedback can be systematically manipulated on top of the visual channel. Various studies supported the positive impact of virtual and augmented reality (VR/AR) as a tool to manipulate and integrate visual and sensorimotor haptic feedback on task performance in a controlled manner (for review; Braun et al. 2018; Kong et al. 2017; Prewett et al. 2012; Kappers 2011). Providing vibro-tactile stimulation in AR also allows more direct comparisons with conditions involving real physical tools than VR because the participant’s normal physical environment remains fully visible during the training. Therefore, the use of haptic feedback in AR might improve performance by making our experience of interacting with objects more realistic. In our own previous study (Jahanian Najafabadi et al. 2022), we showed that immersive VR and AR can provide rich multisensory experiences, especially in combination with vibro-tactile feedback by so-called ‘cybergloves’. We found that the emergence of ownership depended on BS plasticity but not the type of feedback. Emergence of agency was independent of feedback type and BS plasticity (Jahanian Najafabadi et al. 2022).

With this study, we aimed to replicate our previous research on YA with a sample of OA to study the association between the emergence of ownership, agency and plasticity of the BS in tool-use training. We used the same virtual tool-use paradigm as in Jahanian Najafabadi et al. (2022) where participants had to grasp a virtual object with a virtual gripper.

Longo et al. (2010) found that the ability to perceive the size of objects touching the skin is linked to an underlying implicit representation of the body’s shape. Further studies suggested, for several body parts, that the perception of tactile distance between two points on the skin is strongly linked with the BS plasticity (Longo 2020; de Vignemont et al. 2005). Previous studies then employed the tactile distance judgement (TDJ) task as a standard paradigm to assess the plasticity of body representation and relies on the ability of the brain to construct a mental map of the body and its parts (Canzoneri et al. 2013; Miller et al. 2014; Taylor-Clarke et al. 2004). In the current study, to test whether the training with the virtual tool led to a change in the arm representation in the BS, we also used a TDJ task which required participants to estimate the distance between two tactile stimuli on the forearm oriented either along the arm axis in proximodistal orientation or orthogonal to it in mediolateral orientation (Jahanian Najafabadi et al. 2022; Miller et al. 2014, 2017).

After each training condition, the participants answered questionnaires about their subjective feeling of ownership and agency over the virtual tool. To further elucidate the role of visual and tactile feedback in OA, participants were trained in two different conditions. In one condition, participants received only visual feedback where they saw the virtual object and virtual gripper in AR, as well as their real hand and the table. In the other condition, when incorrectly touching the object at either side, vibro-tactile feedback was applied on correctly or incorrectly touching the object through a CyberTouch II glove.

Empirical evidence revealed that the BS is likely to remain plastic in the ageing human brain subject to daily sensorimotor experiences by learning, training or experiences depending on occupation (Reuter et al. 2014; Dinse 2006; Dinse et al. 1997; Kuehn et al. 2018). However, the amount of BS plasticity seems to be reduced as is the capacity for motor learning (Vieluf et al. 2015). We, therefore, expected to replicate our previous findings from YA that training with the virtual tool would likewise lead to a change in the arm representation in OA, but that BS plasticity would be reduced. Moreover, we hypothesized that even OA would be able to gain control over the virtual tool as indicated by the emergence of agency, but that due to impaired motor learning and less BS plasticity, incorporation of tools becomes more challenging for OA compared with YA. Experiences of ownership and agency might be comparatively salient in AR, due to their novelty in comparison with more familiar physical tools but might depend on the type of feedback. We therefore predicted that despite age-related sensory decline, combined visual and tactile feedback, as in real-world settings, would be more effective than visual feedback alone.

## Method

### Participants

For this study, 41 healthy right-handed OA (22 males, 19 females, *M*_age_: 68.92, SD: 4.49) were recruited from a cohort of families living in Bremen-Nord, Germany. Each participant was compensated with 10 Euros per hour. Participants had normal-to-corrected vision with no known history of neurological abnormality or disease, provided informed consent (participation and publication), and were naïve to the experimental hypothesis, acuity and errors. Data of two OA Participants had to be discarded due to sickness during the experiment, and the final analyses utilised data from a total of 39 OA participants. All subsequent analyses’ procedures were approved by the Ethics Committee of the University of Bremen and were in accordance with the principles of the Declaration of Helsinki.

### Study design

**Fig. 1:**
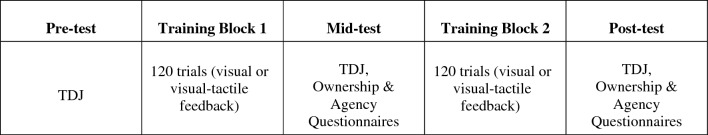
Experimental design

Participants underwent a virtual tool-use training in AR in training blocks with and without vibro-tactile feedback as described in the training section below. The Purdue Pegboard test was used at pre-test to measure unimanual and bimanual finger and hand dexterity of participants. BS was assessed by the TDJ (cf., Jahanian Najafabadi et al. 2022; Miller et al. 2014, 2017) and a tactile localization task (TLT). TDJ and TLT were conducted before training (pre-test), after the training block with the first feedback condition (mid-test) and after the training block under the other feedback condition (post-test). Both ownership and agency for the virtual tool were assessed with questionnaires at mid-test and post-test. Electroencephalography was obtained to record resting-state EEG patterns at all three time points and task-related EEG during both training blocks (cf., Fig. 1) and somatosensory event-related potentials were obtained during the TLT. As the focus of this paper is on changes of the BS and the question of whether the predicted changes in the BS were correlated with the emergence of ownership and agency over the virtual tool, we will report only findings from the TDJ and the ownership and agency assessments. Additionally, behavioural data as an indication of participant’ performance level during virtual tool-use training will be used in our model.

### Virtual tool-use training in AR

**Fig. 2:**
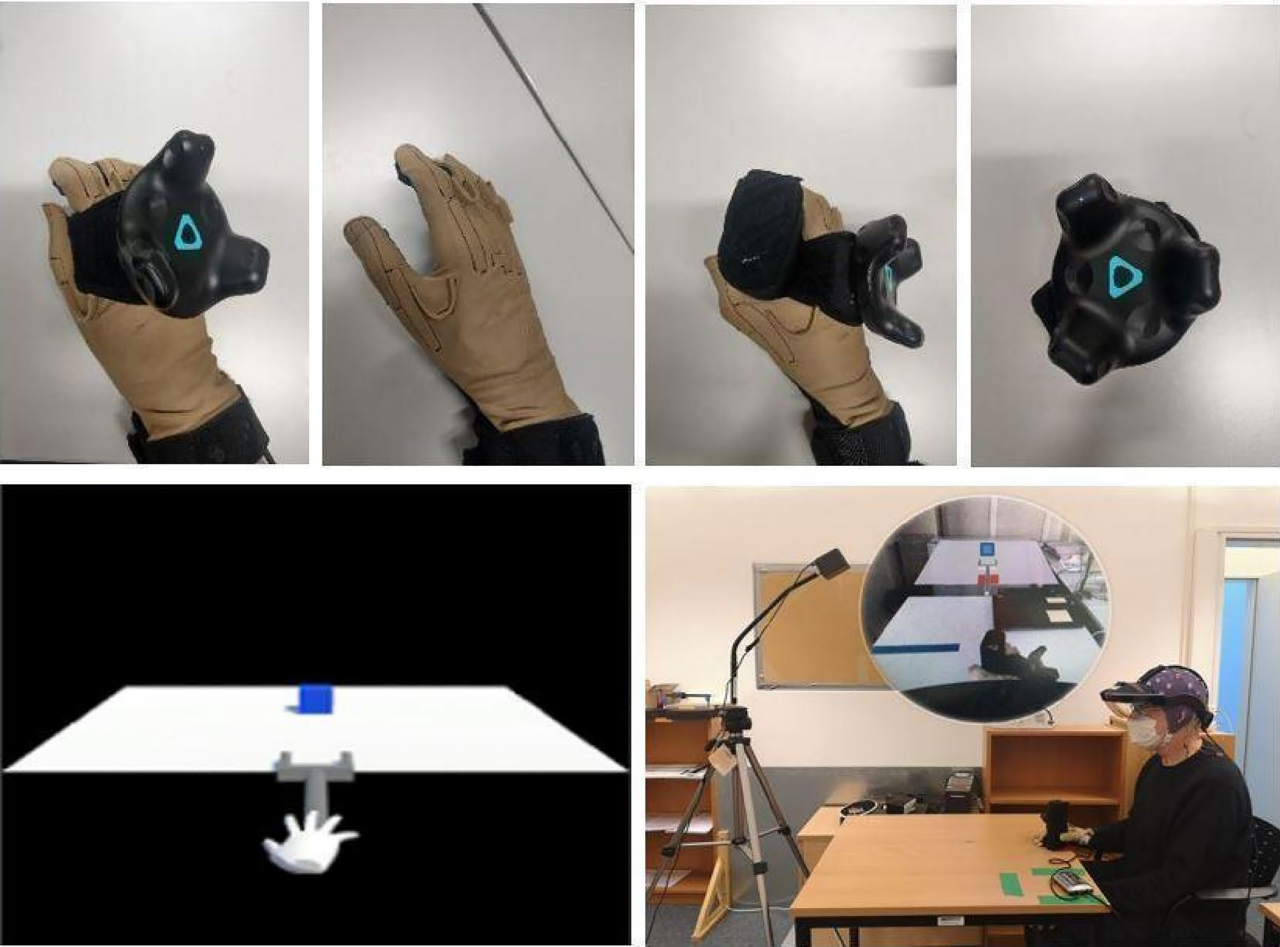
Experimental setup for tool-use training. Top row: CyberTouch-II and Wireless HTC Vive Tracker. Bottom row: Scene view

Participants sat in front of a white table, wearing a Meta2 AR headset (www.metavision.com), which included earphones for receiving verbal instructions. A wireless HTC Vive Tracker 2.0 model KLIM was attached to the back of their right hand. Next, participants donned a special glove (CyberTouch-II, CyberGlove System Inc., 2157 O'Toole Ave, San Jose, USA; cf., Fig. 2) on their right hand. The CyberTouch-II provides fine-grained vibro-tactile feedback on the inside of each finger and the palm. This glove further records the finger movement. The vibrational frequency generated from the CyberTouch-II ranges from 0 to 125 Hz with a total of 6 vibrotactile actuators: one on the inside of each finger, one on the palm. Vibrational amplitude is 1.2 N peak-to-peak at 125 Hz (max). Sensor resolution is 1 degree, sensor repeatability is 3 degrees, and sensor data rate is 90 records/sec.

The experimental AR tool-use training task was implemented in Unity (version 2018.3.8f1) and featured a virtual gripper tool consisting of two parallel legs connected to an elongated stick, and a blue cube as the target object that the participant had to enclose with the legs of the gripper tool (cf., Fig. 2). In addition to the AR environment generated in Unity, participants could see the real surface of the table and their hand. The end of the stick was virtually attached to the hand.

The virtual tool was modelled in Unity in a way that when overlaid with the physical table in physical space, its length equated to about 30 cm in the real reaching space of participants when placed at the starting position in front of the participant. Given a forearm length of 25 cm (flat on the physical table), all cubes in the virtual space could be reached. This estimate is not perfectly precise because the apparent size of the entire scene was influenced by the exact distance of the projection screen to the eyes of the participant, which in turn could be modulated by the tightness of fit for the device. However, these differences were minimal. Furthermore, the relative proportions of the virtual scene (including the tool, the plane and the objects therein) were fixed and thus equally affected by any such (small) variations.

Participants performed two blocks of training, one block with visual and vibro-tactile feedback (VT condition) and one block with only visual feedback (V condition). Each block consisted of 120 trials in two half blocks and the order of blocks was randomised among participants.

During training, to start a trial, participants first had to place their hand at a central starting position before them, as indicated by a red square. Distance to the red square was kept constant. The blue target cube then appeared at different locations in the plane in front of the participants. Participants had to move their hand, and thus, the virtual gripper, towards the object to grasp it. The task was to enclose the virtual object with the gripper without touching either side or moving the gripper into the object. In the condition with tactile feedback, touching the object resulted in vibratory feedback to either the thumb (touched left), the index finger (touched right), or to the palm (touched at front). A trial ended when the object was correctly enclosed by the gripper within 20 s and the participant moved the hand back to the start position for the next trial. Participants were informed in advance that an error would occur if they touched (or moved) inside the cube for more than 2 s or if they touched the cube with the tool’s left or right sides for more than 2 s. Then the trial would fail and end.

After 10 consecutive trials, there were 10 s of rest. After each half block of 60 trials, there was one minute of rest. After the first block (i.e., in the middle of the experiment), approximately 10 min rest were granted. Before each condition started and during the 1-min break after the first 60 trials, participants were alerted about the type of feedback.

Before training, participants performed 20 practice trials to learn how to control the virtual tool by moving their right hand, forwards, backwards, left, or right.

The size of each side of the target cube was 40 × 40 mm^2^. Each training half block of 60 trials started with maximally open gripper jaws (120% of the cube width, i.e., 48 mm^3^). Gripper size was then changed adaptively by decreasing the width of the tool in steps of 0.4 mm in a 3 down / 1 up staircase procedure. This is to approach a stable 79.4% correct performance level over the practice trials (Leek 2001). Gripper size at this performance level thus is directly related to the practice effect (PE) in respective half blocks. Minimum gripper size was 40.4 mm. Each participant performed the same number of trials independently of correct or incorrect trials. The total PE was calculated per block as the relative gripper size at the end of the block compared to the starting gripper size. PE was calculated by subtracting 48 mm^3^ as the starting size from the average value of the last 5 trials divided by 48 mm^3^ [(average value − 48)/48]. Therefore, negative values indicate improved performance.

### Measures of ownership and agency

To measure ownership and agency, we adopted the ownership and agency questionnaire by Zhang and Hommel (2015) (cf. Table 1). Each statement was scored on a 7-point Likert scale (− 3 “strongly disagree” to +3 “strongly agree”). Four mean scores were calculated for statistical analysis by aggregating three questions each: Q1–Q3 were about the experience of perceiving the hand as one’s own hand, i.e., ownership (this variable is abbreviated as BO) and Q7–Q9 were directly associated with the experience of intentional control, i.e., agency (BA). “BO-related” (Q4–Q6) and “BA-related” (Q10–Q12) concerned ownership and agency indirectly (Zhang and Hommel 2015). Scores from Q10–Q12 were reverse-coded, as the corresponding questions are phrased in terms of a loss of control over the tool. According to Kalckert and Ehrsson (2012, 2014), an average score needed to be higher than +1 to indicate the emergence of ownership and agency. Cronbach’s alpha for this study was calculated for each of the four subscales, with all scales demonstrating acceptable to excellent internal consistency in the first measurement (BO: *α* = 0.77, BO-related: *α* = 0.50, BA: *α* = 0.60, BA-related: *α* = 0.85, General scale: *α* = 0.65).

**Table 1:** Statements used in the ownership and agency questionnaire (adapted from Zhang and Hommel 2015)

Variable	Statement
BO	Q1: I felt as if the virtual tool was an extension of my own hand
	Q2: I felt as if the virtual tool was part of my body
	Q3: I felt as if the virtual tool was my hand
BO-related	Q4: It seems as if I had more than one right hand
	Q5: It felt as if my right hand no longer mattered, as if I only needed to sense the virtual tool
	Q6: I felt as if my real hand developed an enhanced sense of virtual touch
BA	Q7: I felt as if I could cause movements of the virtual tool
	Q8: I felt as if I could control movements of the virtual tool
	Q9: The virtual tool was obeying my will and I could make it move just like I wanted it to
BA-related	Q10: I felt as if the virtual tool was controlling my movements
	Q11: It seemed as if the virtual tool had a will of its own
	Q12: I felt as if the virtual tool was controlling me

### Tactile distance judgement (TDJ) task

**Fig. 3:**
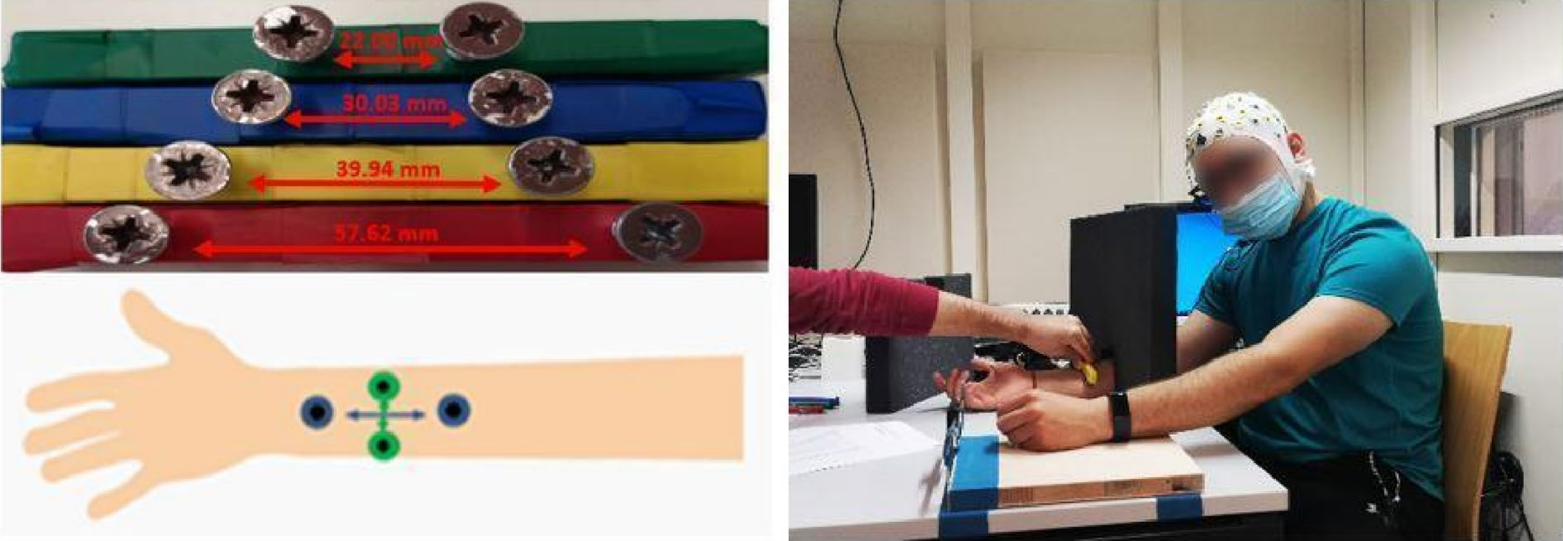
The tactile distance judgement test, with two different orientations (proximodistal, mediolateral) applied to the forearm

We applied a TDJ task as pre-test, mid-test and post-test (adapted from Miller et al. 2014, 2017). Wooden blocks were prepared with four sample pairs of screws, each with different distances between them. The screws had round tips with 9 mm diameter. For TDJ testing parallel to the arm axis (“proximodistal” alignment), 3 sample pairs with distances of 57.62, 39.94, and 30.03 mm were prepared. For TDJ orthogonal to the arm axis (“mediolateral”), because of the anisotropy of RFs, the sample with the largest distance (57.62 mm) was replaced by a sample with a smaller distance of 22.00 mm. For each trial, one sample was applied in pseudorandomized order to the right forearm onto the mediolateral or proximodistal orientation (cf., Fig. 3). Each trial lasted approximately 1 s. Each sample was presented 5 times, resulting in 30 trials in total (15 proximodistal and 15 mediolateral; 5 trials each per 3 distances per orientation).

In previous studies (Miller et al. 2014, 2017), participants were instructed to report verbally to the experimenter whether they perceived the distance between the two stimuli on the skin as shorter or longer than a reference in the forehead. In this study, we required participants to report absolute estimates of the distances. This is in accordance with other studies collecting absolute estimates such as verbal estimates (Longo and Golubova 2017; Fiori and Longo 2018), adjustments of a visually perceived line (Tamè et al. 2021), or kinaesthetic matching of the distance between two fingertips (Keizer et al. 2011; Knight et al. 2014).

Participants were instructed to indicate the perceived distance between the centres of the screws using a digital calliper (analogue scale). The calliper consisted of two steel legs fixed to a piece of wood attached to the table close to the participant’s left hand. The distance between the legs could be easily adjusted by the participant. Right after each TDJ stimulus, participants were asked to use their left hand to report the perceived distance by adjusting the distance between the calliper legs and the distance was noted by the experimenter based on the mm scale displayed on the calliper monitor (see Fig. 3). Importantly, participants were prevented from seeing the stimuli presented to their forearm by putting an obstacle between their eyes and the right forearm throughout the TDJ test. Therefore, they had no visual information about the real distances, and whether the pairs of stimuli were administered on proximodistal or mediolateral orientation.

The judgement error was used as an indicator of perceived arm length and calculated as the difference between the reported distance and the actual distance that was presented (error = estimated distance–real distance). Positive values thus indicated an overestimation and negative values an underestimation of the distance. Estimation errors were averaged over the five trials per condition (orientation and distance) and calculated separately for pre-test, mid-test and post-test. A decrease in the distance judgment would indicate that after tool-use training, the virtual tool was appended to the sensorimotor representation of the arm within the extent of the existing BS, i.e., the somatotopic cortical representation. As a consequence, the arm would become perceptually shorter, and different locations on the proximodistal orientation of the forearm would be perceived as closer together.

### Data analysis and statistics

The dataset that was generated and analysed during the current study will be made available on publication in an Open Science Framework repository on OSF.io. Inferential statistics were performed with R (R Core Team 2021) and the Jamovi software environment version 2.2.5.6.2 (The Jamovi project 2021). All relevant R packages (v4.1.1; RStudio v1.4.1717) and related references are listed in the supplement. If not stated differently, *p* < 0.05 were considered as significant, and p values < 0.10 as marginally significant throughout the report.

## Results

### Baseline asymmetry in TDJ error

**Fig. 4:**
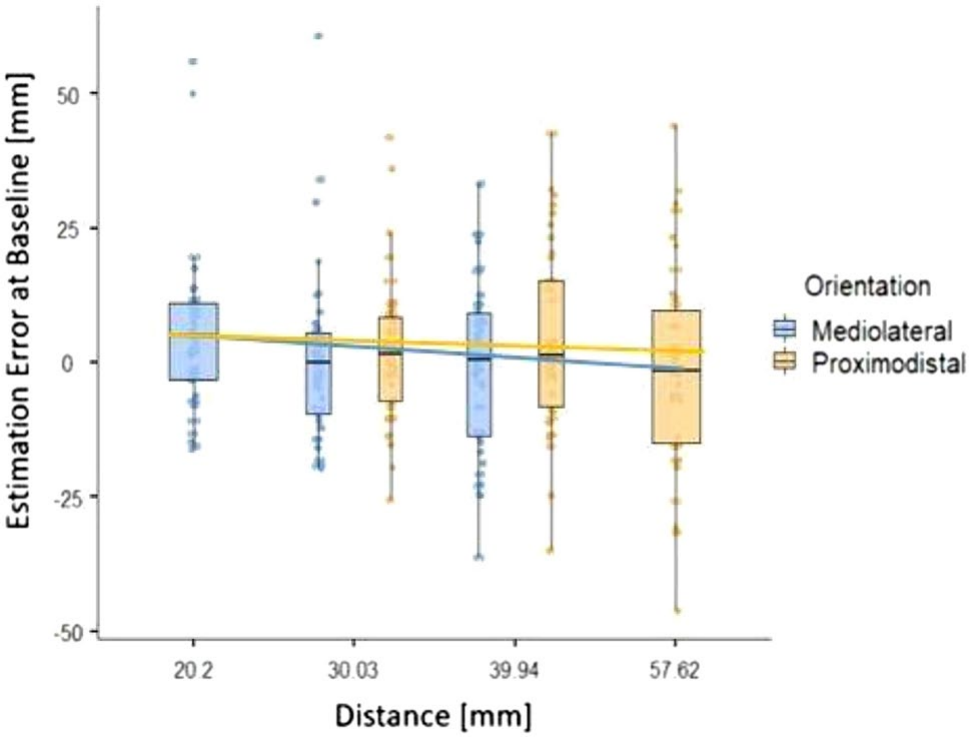
TDJ Estimation Errors in mm at baseline dependent on stimulus Distance and Orientation. Boxes represent medians and interquartile ranges. Whiskers show largest values within 1.5 times the interquartile ranges. Lines show regression lines based on estimates derived from the linear model

First, we examined baseline differences in Estimation Errors for different Orientations and Distances. Figure 4 displays TDJ Estimation Errors at baseline in mm for different Distances and Orientations. Results indicate that the Estimation Errors were reduced with increasing Distances in both proximodistal and mediolateral Orientations.

To confirm baseline asymmetries of distance judgement errors in the TDJ, we fitted a general linear model (GLM) to predict Estimation Error at baseline with Orientation (proximodistal or mediolateral) and Distance in mm. The model's explanatory power is weak but marginally significant (*R*^2^ = 0.016, adj. *R*^2^ = 0.009, *F*(3464) = 2.55, *p* = 0.055; cf. Table 2). The model's intercept is at 1.24 (95% CI [− 0.5, 2.99]) indicating neither under nor overestimation. Within this model, the effect of Distance was significantly negative (*t*(464) = − 2.77, *p* = 0.006), indicating reduced Estimation Errors with increasing Distance. The effect of Orientation (*t*(464) = 1.4, *p* = 0.163) and the interaction effect of Orientation and Distance were positive but not significant (*t*(464) = 1.02, *p* = 0.31).

**Table 2:** Effects of stimulus Orientation and Distance on estimation errors in the TDJ

	Estimate	*SE*	*t*	*p*
(Intercept)	1.245	0.89	1.40	0.163
Orientation	2.66	1.78	1.49	0.136
Distance	− 0.22	0.079	− 2.77	0.006
Orientation × Distance	0.163	0.159	− 1.02	0.306

*df* = 464, Multiple *R*^2^ = 0.016, Adjusted *R*^2^ = 0.009, *F*(3464) = 2.55; *p* = 0.055

### Practice effect and role of feedback type

**Fig. 5:**
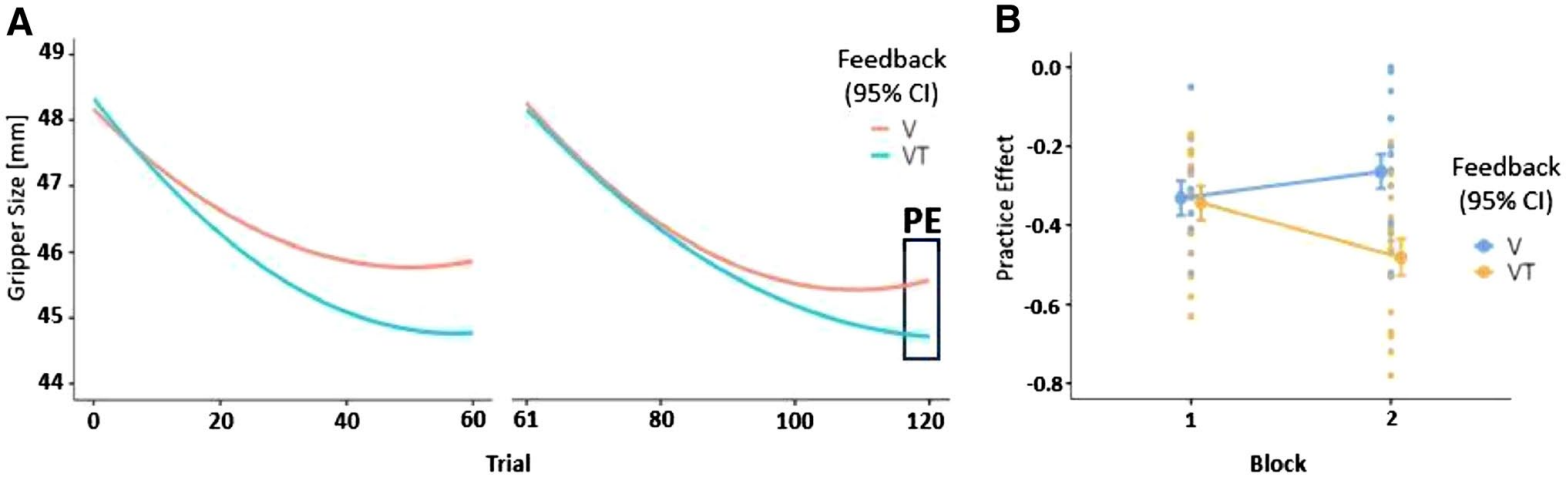
**A** Gripper size during training Blocks with VT versus V Feedback conditions. The square on the right denotes the last 5 trials which were used to calculate the practice effect (PE). **B** PE over the course of the experiment for VT versus V Feedback conditions depending on Block number. Negative values indicate reduced gripper size and better performance

The final performance level as approached by the staircase procedure during training Blocks was lower for the VT Feedback compared to the V Feedback condition (cf., Fig. 5). This indicates that VT Feedback was more effective. To quantify and statistically test the effect of Feedback condition on PE, GLM analysis with PE as a dependent variable and Feedback type (V or VT) and Block number (first or second training block) as factors were conducted.

Results revealed a significant effect of Feedback (*F*(1185) = 26.16, *p* < 0.001) and an interaction effect of Block x Feedback on PE (*F*(1185) = 26.76, *p* < 0.001; cf., Table 3). Further, the model’s explanatory power is strong and significant (*R*^2^ = 0.205, adj. *R*^2^ = − 0.193, *F*(3185) = 15.95, *p* < 0.001*;* cf., Table 3). The model’s intercept is at -0.355 (95% CI [− 0.38, − 0.33]) indicating a reduction in gripper size on average.

**Table 3:** GLM for practice effect as dependant variable and Block x Feedback as factors

	SS	*df*	*F*	*p*	*η*^2^
Model	1.1321	3	15.95	< 0.001	0.205
Block	0.0581	1	2.46	0.119	0.011
Feedback	0.6191	1	26.16	< 0.001	0.112
Block × feedback	0.4913	1	20.76	< 0.001	0.089
Residuals	4.3782	185			
Total	5.5103	188			

A post hoc t-test revealed that PE was significantly stronger (more negative, indicating more reduction in gripper size) for the VT than for the V Feedback condition (*t*(185) = − 5.11; *p* < 0.001, cf., Fig. 5). This difference was mainly driven by the difference between VT and V in the second practice Block (t(185) = − 4.56, *p* < 0.001).

### Effects of tool-use training on TDJ error

To test our assumption that training would affect TDJ error in the proximodistal Orientation, we followed a two-step procedure. We first analysed the effects of Distance and baseline error on post-test error. The residuals of this analysis represent the variance in error after training that is not explained by baseline and Distance and was then used for analysing the Orientation effect on TDJ error after training. This allowed us to evaluate Orientation effects on Estimation Errors after training regardless of the different distances used for both Orientations. Without any training effect, residuals should be around zero. Negative residuals would indicate reduced estimation errors. A tendency for reduced estimation errors in proximodistal as compared to mediolateral Orientation would result in more data points laying below the diagonal.

**Fig. 6:**
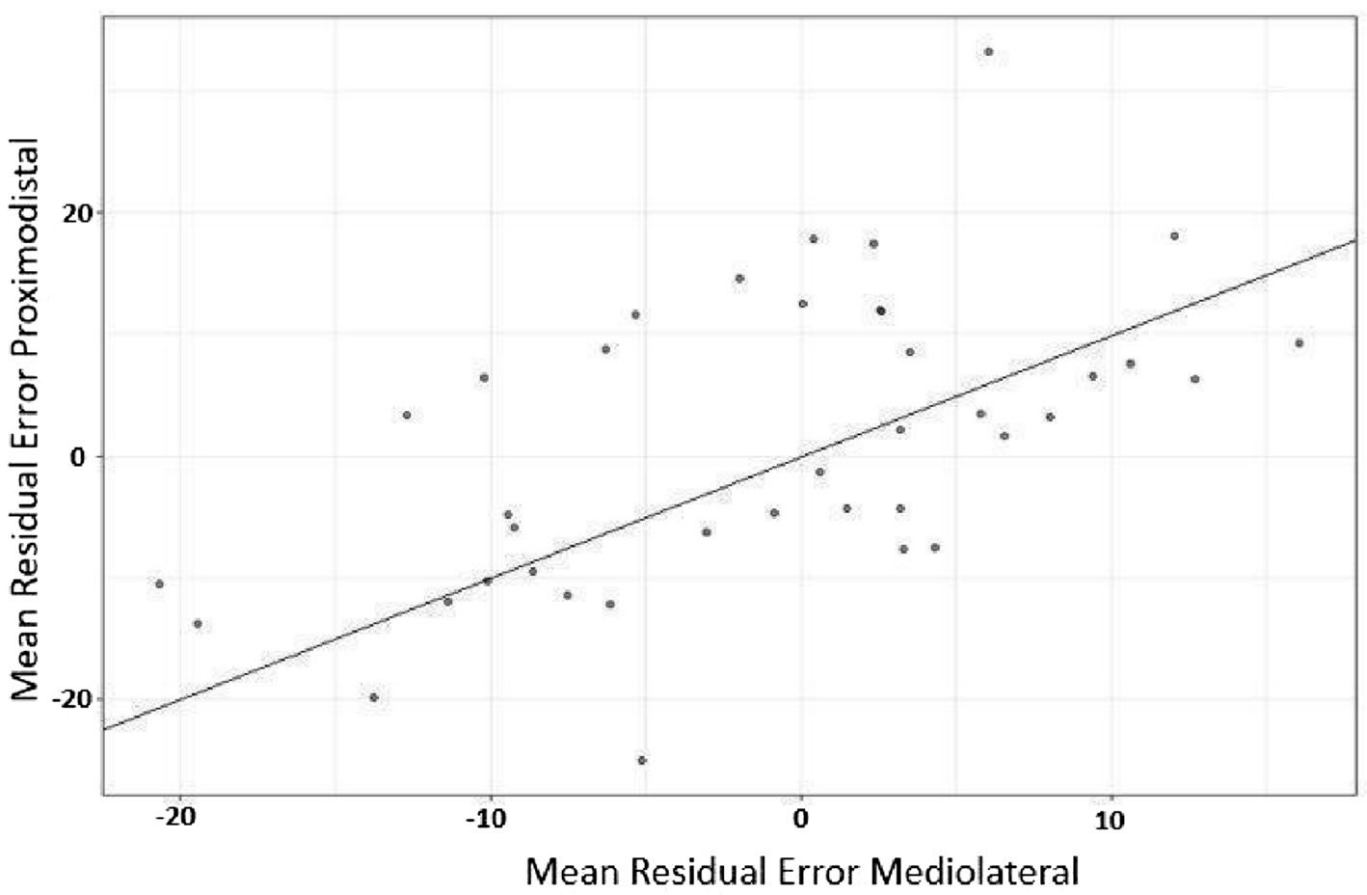
Mean Residual Estimation Errors per individual for proximodistal and mediolateral Orientations. The solid line represents the diagonal. Points above or below the diagonal indicate no differences in average residuals for proximodistal and mediolateral Orientations in the same individual

**Fig. 7:**
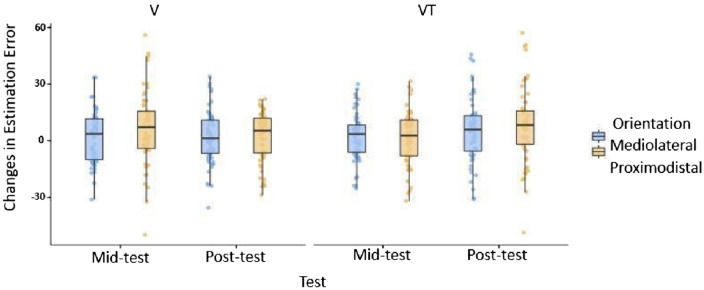
Changes in Estimation Error at mid-test and post-test for mediolateral and proximodistal Orientations and for Feedback conditions. Boxes represent medians and interquartile ranges. Whiskers show largest values within 1.5 × the interquartile ranges

As illustrated in Fig. 6, there was no tendency that residuals were larger or smaller for the proximodistal Orientation as compared to the mediolateral Orientation. As the assumption of normality was violated (Shapiro–Wilk *W* = 0.99, *p* = 0.026), we performed a Wilcoxon Rank test to compare both Orientations. Result revealed that residuals for the mediolateral Orientation (median = -1.66, SE = 0.75) were not significantly different than those for the proximodistal Orientation (median = 0.910, SE = 0.951; *W*(233) = 15,214, *p* = 0.157; cf., Fig. 7).

There was also no significant Orientation effect on the residuals when taking Feedback and Test (mid-test, post-test) into account. We fitted a GLM to predict the Residual Estimation error with Orientation, Test and Feedback. The model’s explanatory power is very weak and not significant (*R*^2^ = 0.013, adj. *R*^2^ = − 0.001, *F*(7460) = 0.92, *p* = 0.492*;* cf., Table 4). The model’s intercept is at 0.011 (95% CI [-1.17, 1.20]). Contrary to our prediction, no significant main or interaction effects could be revealed within this model (*p* = 0.985).

**Table 4:** Effects of Orientation, Test and Feedback on changes in Residual Estimation Error after training

	Estimate	*SE*	*t*	*p*
(Intercept)	0.011	0.61	0.01	< 0.985
Orientation	1.915	1.211	1.58	0.115
Test	1.444	1.211	1.192	0.234
Feedback	1.215	1.211	1.003	0.316
Orientation × Test	− 0.193	2.423	− 0.079	0.937
Orientation × Feedback	− 1.482	2.423	− 0.612	0.541
Test x Feedback	1.787	2.423	0.737	0.461
Orientation × Test × Feedback	4.064	4.845	0.838	0.402

*df* = 460, Multiple *R*^2^ = 0.01, Adjusted *R*^2^ = − 0.001, *F*(7460) = 0.92, *p* = 0.492

### Body ownership and body agency after virtual tool-use

**Fig. 8:**
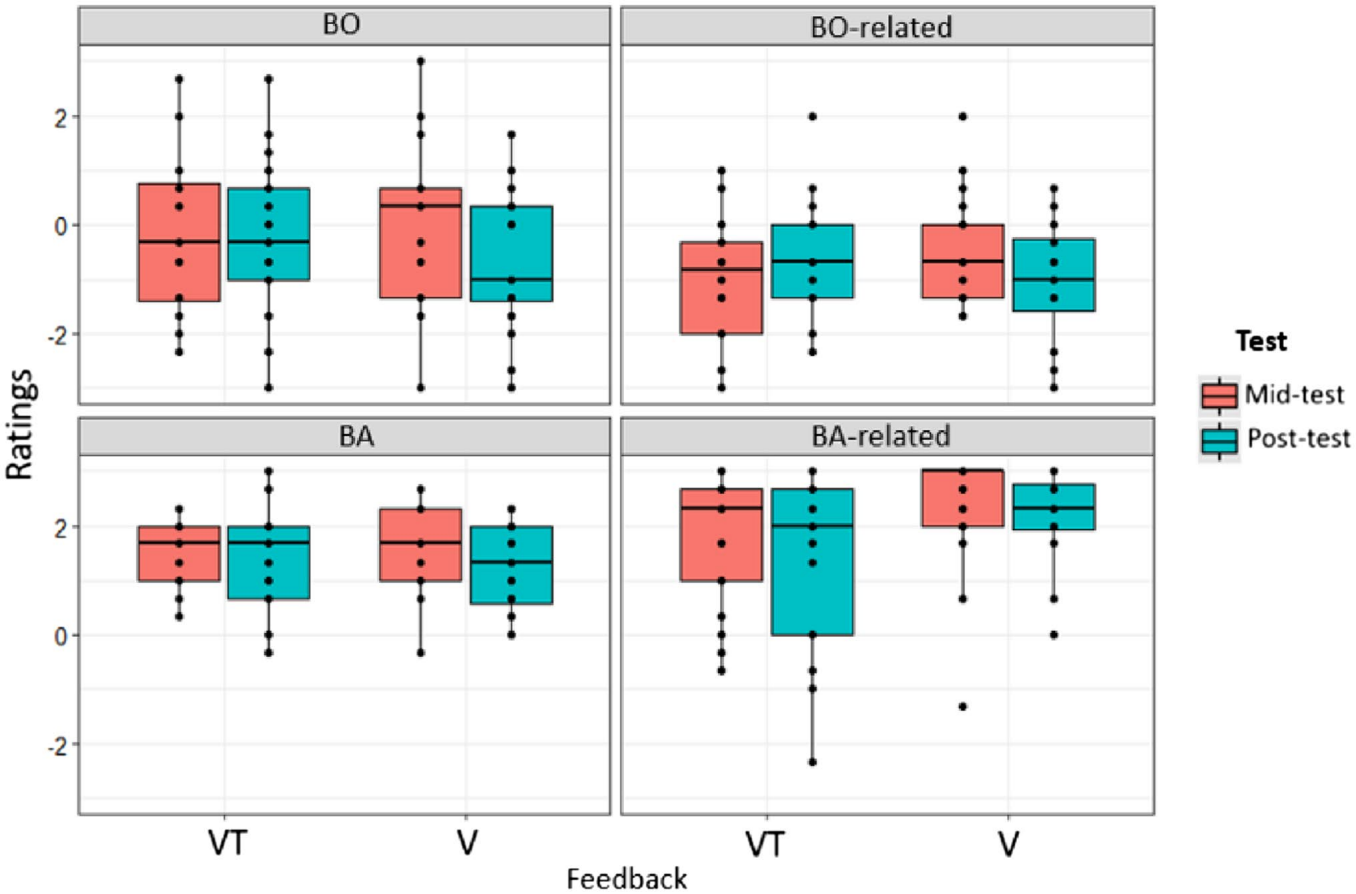
Ratings of BO, BO-related, BA, and BA-related subscales depending on Feedback condition and Test. Boxes represent medians and interquartile ranges. Whiskers show largest values within 1.5 times the interquartile ranges

Descriptive analysis of ownership and agency revealed values of − 0.3 ± 0.07 and − 0.8 ± 0.05 for BO and BO-related, and 1.4 ± 0.4 and 1.9 ± 0.6 for BA and BA-related, respectively (means and *SE*). Thus, only mean values for BA and BA-related, but not BO and BO-related, were above 1 as the threshold suggested by Kalckert and Ehrsson (2012, 2014). Figure 8 illustrates these findings separated by Feedback condition and Test.

To analyse whether the emergence of Ownership and Agency was dependent on Feedback, Test, BS plasticity or PE, we performed a three-step linear regression separately for Ownership and Agency ratings. In the first step, we analysed whether ratings were predicted by Feedback and Test or the interaction of Feedback and Test (model 1). In the second step we added PE (model 2) and in the third step we added TDJ Estimation Error to the regression model (model 3; cf. Table 5).

**Table 5:** Model fit parameters of the three linear regression models for Ownership and Agency variables

	Model 1						Model 2						Model 3					
	*R*	*R*^2^	Adjusted *R*^2^	*F*	RMSE	*p*	*R*	*R*^2^	Adjusted *R*^2^	*F*	RMSE	*p*	*R*	*R*^2^	Adjusted *R*^2^	*F*	RMSE	*p*
BO	0.20	0.04	0.02	2.59	1.32	0.054	0.21	0.04	0.02	1.94	1.32	0.105	0.23	0.05	0.02	2	1.31	0.080
BO-related	0.31	0.09	0.08	6.68	1.07	< 0.001	0.32	0.10	0.08	5.24	1.06	< 0.001	0.32	0.10	0.07	4.20	1.06	< 0.001
BA	0.11	0.01	− 0.003	0.77	0.813	0.508	0.21	0.04	0.02	2.17	0.79	0.074	0.22	0.05	0.02	1.89	0.79	0.097
BA-related	0.26	0.07	0.05	4.60	1.21	0.004	0.27	0.07	0.05	3.73	1.21	0.006	0.27	0.07	0.05	2.97	1.21	0.013

**Fig. 9:**
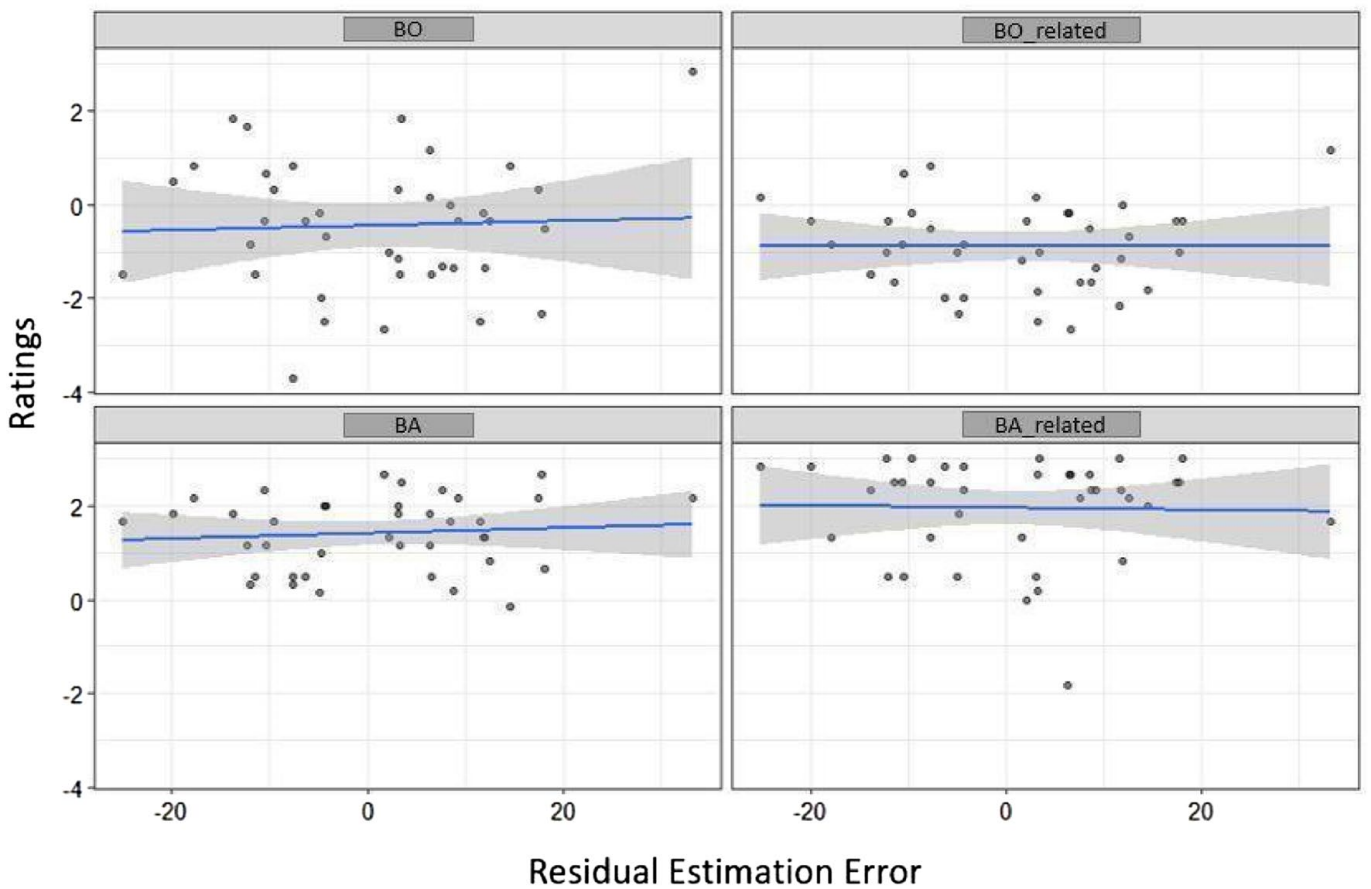
Associations between Residual Estimation Error for proximodistal Orientations in mm and Ownership and Agency ratings. Solid lines and shaded areas represent linear regression lines and 95% confidence intervals

**Fig. 10:**
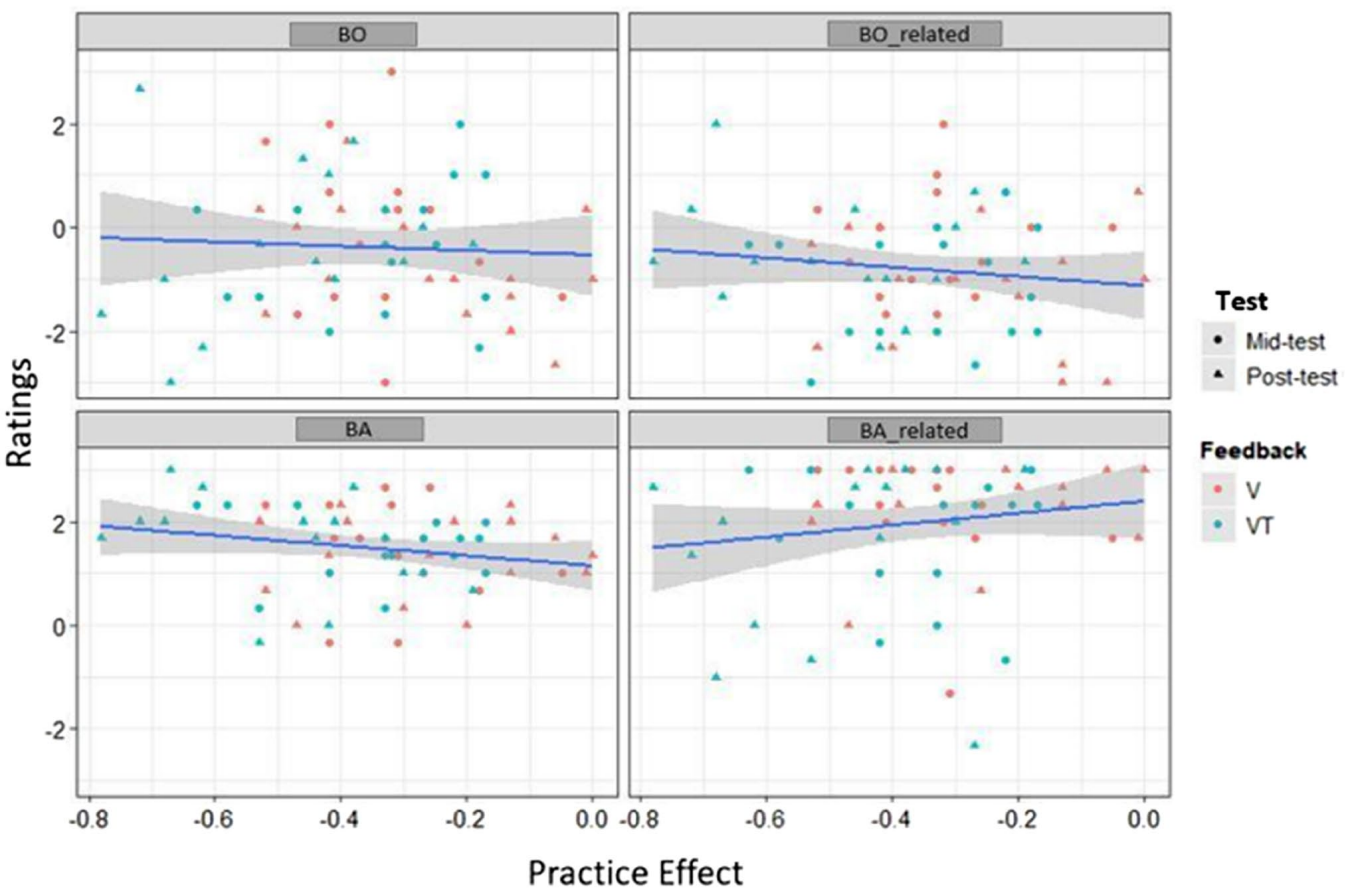
Associations between Practice Effect during virtual tool-use training and Ownership and Agency ratings dependent on Test and Feedback. Solid lines and shaded areas represent linear regression lines and 95% confidence intervals

For BO, model 1 was marginally significant (*R*^2^ = 0.04, adj. *R*^2^ = 0.02; *F*(3185) = 2.59, *p* = 0.054; cf., Table 5) and revealed significant Feedback (*p* = 0.067), Test (*p* = 0.009), and Feedback x Test interaction effects (*p* = 0.012; cf., Table 7). Neither adding PE in model 2 (Model 2 vs. Model 1:* p* = 0.857; cf., Tables 5 and 6) nor Residual Estimation Error in model 3 (Model 3 vs. Model 2:* p* = 0.142) improved the initial model (i.e., increased explained variance or reduced RMSE) and PE and Estimation Error were not revealed as predictors for BO. The interaction effect in model 1, between Feedback and Test, indicates that BO rating decreased in the post-test when V followed the VT condition but not vice versa (cf. Table 7, and Figs. 9 and 10).

**Table 6:** Model comparison of the three linear regression models for Ownership and Agency variables

Comparison	BO			BO-related			BA			BA-related		
	Δ*R*^2^	*F*	*P*	Δ*R*^2^	*F*	*P*	Δ*R*^2^	*F*	*P*	Δ*R*^2^	*F*	*P*
Model 1 vs. 2	0.00	0.03	0.858	0.01	0.91	0.340	0.03	6.28	0.013	0.01	1.095	0.297
Model 2 vs. 3	0.01	2.17	0.142	0.00	0.17	0.679	0.01	0.79	0.373	0.00	0.026	0.871

**Table 7:** Linear regression for Ownership and Agency variables as dependent variables, Feedback and Block as factors and Residual Estimation Error and Practice Effect as covariates

Independent variables	BO			BO-related			BA			BA-related		
	Model 1	Model 2	Model 3	Model 1	Model 2	Model 3	Model 1	Model 2	Model 3	Model 1	Model 2	Model 3
Intercept	− 0.020 (0.192)	− 0.058 (0.286)	− 0.029 (0.285)	− 0.396* (0.156)	− 0.560* (0.231)	− 0.552* (0.232)	1.542*** (0.119)	1.221*** (0.174)	1.231*** (0.174)	2.33*** (0.177)	2.536*** (0.262)	2.533*** (0.263)
Feedback (VT-V)	− 0.50* (0.271)	− 0.50* (0.27)	− 0.508* (0.271)	− 0.749*** (0.220)	− 0.755*** (0.220)	− 0.756*** (0.220)	− 0.0644 (0.168)	− 0.077 (0.165)	− 0.079 (0.165)	− 0.564* (0.250)	− 0.556* (0.251)	− 0.555* (0.252)
Test	− 0.708** (0.270)	− 0.70** (0.272)	− 0.722** (0.273)	− 0.767*** (0.219)	− 0.734*** (0.222)	− 0.738 (0.222)	− 0.203 (0.167)	− 0.138 (0.167)	− 0.146 (0.166)	− 0.129 (0.248)	− 0.170 (0.251)	− 0.167 (0.252)
Feedback × Test	0.971** (0.387)	0.948* (0.409)	0.992* (0.409)	1.388*** (0.314)	1.288*** (0.331)	1.297*** (0.33)	0.302 (0.239)	0.104 (0.249)	0.120 (0.25)	− 0.140 (0.356)	− 0.015 (0.375)	− 0.020 (0.377)
PE		− 0.114 (0.637)	0.018 (0.641)		− 0.493 (0.516)	− 0.462 (0.521)		− 0.970* (0.387)	− 0.921* (0.391)		0.611 (0.584)	0.598 (0.591)
Residual Estimation Error			0.009 (0.006)			0.002 (0.005)			0.003 (0.004)			− 0.001 (0.006)

Beta Coefficients and Standard errors (in parenthesis) are reported. ***p < 0.001, **p < 0.01, *p < 0.05

For BO-related, model 1 (cf., Table 5) was statistically significant (*R*^2^ = 0.09; adj. *R*^2^ = 0.08; *F*(3185) = 6.68; *p* < 0.001) and revealed significant effects of Feedback (*p* < 0.001) and Test (*p* < 0.001), and Feedback × Test interaction effects (*p* < 0.001; cf., Table 7). The interaction effect demonstrated that ratings where higher for V Feedback condition than the VT condition in the mid-test but not the post-test. Adding PE in model 2 did not improve the initial model (*p* = 0.340) and an effect of PE was not revealed (beta = − 0.493*, SE* = 0.516, *p* = 0.340). Further adding Residual Estimation Error in model 3 did also not improve the model (*p* = 0.679) and also Estimation Error was not significant (beta = 0.002*, SE* = 0.005, *p* = 0.679).

For BA, model 1 was not significant (*R*^2^ = 0.01, adj. *R*^2^ = − 0.003; *F*(3185) = 0.77, *p* = 0.508). Adding PE in model 2 marginally significantly improved the initial model (*p* = 0.013; cf., Table 6) and model 2 became marginally significant (*R*^2^ = 0.04, adj. *R*^2^ = 0.02; *F*(4184) = 2.17, *p* = 0.074). PE significantly predicted BA (beta = − 0.970*, SE* = 0.387, *p* = 0.013). Larger reduction in gripper size (more negative PE values) was associated with higher BA ratings (cf., Fig. 10). Adding Residual Estimation Error in model 3 did not further improve the model (*p* = 0.373) and no significant effect of Residual Estimation Error was revealed (beta = 0.003, SE = 0.004, *p* = 0.372).

For BA-related, model 1 was significant (*R*^2^ = 0.07, adj. *R*^2^ = 0.05; *F*(3185) = 4.60, *p* = 0.004, cf., Table 5) and a significant effect of Feedback was revealed (beta = − 0.564, SE = 0.250, *p* = 0.025). BA-related was lower in the VT Feedback condition compared to the V Feedback condition (*p* = 0.025, cf., Table 7). Neither adding PE in model 2 (*p* = 0.297, cf., Table 6) nor Estimation Error in model 3 (*p* = 0.871) improved the model and both variables did not predict BA-related.

## Discussion

In this study, we aimed at replicating in a sample of OA previous findings from YA (Jahanian Najafabadi et al. 2022) which suggested that training to control a virtual tool in AR results in emergence of ownership and agency over the virtual tool and that ownership is associated with BS plasticity, as revealed by reduced estimation errors in a tactile distance judgement task. While we could confirm that OA were able to learn to control the tool as well as YA and develop a sense of agency, against our expectations, no BS plasticity and no emergence of body ownership was revealed.

### Older adults are well able to learn controlling the virtual tool

Older adults were able to learn to control the virtual tool. The practice effect was of a similar magnitude as previously described for YA. Results showed that participants performed significantly better and faster during virtual tool-use training in both blocks with VT and V feedback conditions. However, on average, they had a steeper learning slope and greater improvement in their performance in the block with VT feedback condition. Thus, in accordance with our behavioural findings, differences in learning effect between the two blocks are explained by types of feedback they received. In this line, as in YA, the practice effect was stronger in the VT feedback condition as compared to the V condition indicated by larger reduction in gripper size (more negative PE values). This highlights the importance of multisensory integration in tool-use learning in older age, putting forward the idea that OA perform significantly better when two sensory stimuli are integrated or combined, rather than relying on only one modality (e.g., vision) without any tactile or auditory information (Wahn et al. 2020). Our results are further supported by Mahoney and colleagues who reported that around 75% of OA showed faster performance in visual-somatosensory testing conditions compared with either vision or somatosensory conditions alone (Mahoney et al. 2014). Thus, OA might benefit more from multisensory inputs compared to unisensory ones, although integration of visual-tactile modalities is one of the major difficulties occurring with age (for review; Freiherr et al. 2013; Mahoney et al. 2014; Costello and Bloesch 2017).

### Virtual tool-use training did not induce changes in the body schema of older adults

Previous studies supported the typical and generalised pattern across young participants that either physical or virtual tools became incorporated into the existing BS of the forearm enlarging the PPS (Jahanian Najafabadi et al. 2022; Miller et al. 2014, 2017; Cardinali et al. 2012). In our study, despite the similar strength of the practice effect as in YA, while direct statistical comparison is not possible, OA did not reveal BS plasticity. This is in line with a study by Costello et al. (2015), similarly showing that OA did not exhibit any changes in estimation error after tool-use. Their study additionally revealed an overestimation of target distances beyond the PPS both during the use of a tool and during pointing to a target with their hand. Besides age-related decline in representational plasticity, it might also be that due to a decline in visuo-spatial processing and mental transformations and thus impaired multisensory information less alterations of the BS are induced (Devlin and Wilson 2010; Kochunov et al. 2005; Lehmbeck et al. 2006; Makin et al. 2007). Previous researchers (Devlin and Wilson 2010; Ghafouri and Lestienne 2000) suggested that, to form a stable spatial representation of the body, older people, compared to younger ones, show less flexibility when integrating new sensory information. BS plasticity in OA was independent of feedback conditions. While from previous studies, one would expect that multisensory integration is required for recalibrating bodily information by synchronous visual inputs (Costello and Bloeesch 2017), one might speculate that such multisensory integration is affected in OA due to decrease in unisensory process (Stein and Stanford 2008). Altered sensory representations with less precise body and space representations (Sorrentino et al. 2021) and reduced processing speed (Costello and Bloeesch 2017) might contribute to this effect, e.g., through unmatched timing. Reduced multisensory integration might also be related to reduction in attentional resources and attentional capacities in one specific modality (Hugenschmidt et al. 2009). Further, decreased attentional capacities in OA could also explain their slower sensory processing (Riis et al. 2009).

Additionally, the ability of OA to perform visuo-motor remapping may be impaired, as older adults do not experience changes in their perceived distance to targets as a result of tool-use as young adults do (Caçola et al. 2013; Costello et al. 2015; Kuehn et al. 2018).

Another explanation for the fact that we could not confirm larger TDJ errors in proximodistal as compared to mediolateral directions could be based on age-related changes in somatosensory cortical processing. OA have larger receptive fields likely caused by reduced intracortical inhibition. As a consequence, neuronal responses may become more broadly tuned and receptive fields larger. Assuming that anisotropy of receptive fields is based on intracortical inhibition, receptive fields should get larger particularly in mediolateral direction, resulting in increased estimation errors (Lenz et al. 2012; Pleger et al. 2016).

 Tanaka (2021) revealed that internal body models represented by the BS and BI are at the core of motor learning. While the BI is necessary for the initial phase of motor learning, the BS is a prerequisite for the advancement of motor learning in the next phases by incorporation of new ways of movement that updates constantly and unconsciously. The repetitive movement of body parts during motor practice leads to the improvement of performance by requiring the learner to develop a new set of processes associated with practice, consciousness and mental processes when doing the task. Therefore, given that BS plasticity was not observed in our study in spite of motor learning, the question still remains whether BS plasticity is a prerequisite for any type of motor learning or vice versa.

### Changes in ownership and agency were not related to altered body schema

As expected, participants developed a modified sense of agency during training which was predicted by the practice effect but not BS plasticity. Findings suggest that even in OA, agency may strongly relate to improvement in tool-use, rather than depending on changes in plastic reorganisation of the sensorimotor representation. Previous research in YA indicated that haptic feedback influences the BR and our interaction with virtual objects while grasping (Krogmeier et al. 2019). For example, haptic feedback in VR might improve performance by making our experience of interacting with objects more realistic (Kappers, 2011). However, although the practice effect was stronger after VT feedback as compared to V feedback and agency increased with practice effect, no clear dependency of agency on feedback condition could be revealed. Rather, BA-related ratings were higher in the V condition as compared to the VT condition indicating that other factors also must be involved not assessed in this study. Probably, in case of conflicting information, OA more relied on the visual input to control the virtual tool. Another explanation might be that agency ratings were very high in all conditions and thus a ceiling effect might have masked the effect of feedback condition in agency ratings.

However, against our hypothesis and contrary to previous studies that revealed decreased levels of subjective feeling of ownership experience in the RHI in OA (Kállai et al. 2017; Graham et al. 2014), our results revealed no emergence of ownership during tool-use training. Further research also revealed lower agency and ownership in OA compared to YA and concluded that OA generally experience an attenuated sense of agency and ownership (Cioffi et al. 2017). As for agency, this cannot be confirmed by our results. This touches upon the idea that OA might be less susceptible to the RHI but not to the embodiment of tools in general (Weser and Proffitt, 2021) indicating that RHI compared with tools incorporated into the body and not extending it. We speculate that OA may need longer training with virtual tools to induce BS plasticity and a sense of ownership and that tasks must rely even more on multisensory integration. Combined, more work is still needed in order to fully understand the underlying mechanisms and the association of ownership, and agency with changes in BS plasticity and BI.

## Limitations

Some limitations of our study need to be addressed: Results cannot be directly compared with our previous study in YA as these are two separate studies using the same experimental design but at different physical laboratories and locations. Another limitation is that in our study, visual channels always existed on top of haptic feedback and this modality couldn’t be controlled during the training. This only allowed us to control and evaluate the contribution of vibrotactile feedback. Therefore, we suggest that future studies should take the advantage of AR to study malleability of the sensorimotor BS in new experimentally controlled and ecologically valid paradigms, including modulation of the visual modality.

## Conclusions and outlook

We conclude that a sense of agency may strongly relate to improvement in tool-use in OA dependent on the PE but independent of alterations in the BS, while ownership did not emerge due to a lack of BS plasticity.

We suggest that the future studies should take advantage of AR methods to easily isolate and manipulate sensory information. This could very much contribute to our understanding of age differences in the emergence of ownership and agency during tool-use training and whether training with different sensory modalities would improve the learning outcome, or reduce the effect due to a conflict between different sensory modalities. Further understanding would also be useful for rehabilitation programs, especially for OA, or the facilitation of motor learning in clinical patients.

## Supplementary Information

Below is the link to the electronic supplementary material.

Supplementary file1 (DOCX 19 KB)

## Data Availability

The dataset that was generated and analysed during the current study will be made available on publication in an Open Science Framework repository on OSF.io.
