# Supplementary material for: Tool-use training in augmented reality: plasticity of forearm body schema does not predict sense of ownership or agency in older adults
Source: Exp Brain Res. 2023 Jun 12;241(7):1739–56. doi: 10.1007/s00221-023-06645-2 (PMC10348985; doi:10.1007/s00221-023-06645-2)
Supplement: Supplementary file 1 — Supplementary file1 (DOCX 19 KB) [file 221_2023_6645_MOESM1_ESM.docx]

**Tool-use training in augmented reality: Plasticity of forearm body schema does not predict sense of ownership or agency in older adults**

Amir Jahanian Najafabadi^*1,2^, Dennis Küster^3^, Felix Putze^3^, Ben Godde^2^

^1^Department of Cognitive Neuroscience, Bielefeld University, 33501 Bielefeld, Germany

^2^School of Business, Social and Decision Sciences, Constructor University Bremen, 28759 Bremen, Germany
 ^3^Department of Computer Science, University of Bremen, 28359 Bremen, Germany

*Corresponding author: [amir.jahanian@uni-bielefeld.de](mailto:amir.jahanian@uni-bielefeld.de)

**Supplement**

**R packages used**

- base (R Core Team 2021)
- car (Fox and Weisberg 2019)
- gamlj (Gallucci 2021)
- ggplot2 (Wickham 2016)
- grateful (Rodríguez-Sánchez and Hutchins 2020)
- jmv (Selker et al. 2021)
- jmvcore (Love 2021)
- readxl (Wickham and Bryan 2019)
- report (Makowski et al. 2021)
- sjlabelled (Lüdecke 2021a)
- sjmisc (Lüdecke 2018)
- sjPlot (Lüdecke 2021b)
- tidyverse (Wickham et al. 2019)
- corrplot (Wei & Simko 2021)
- dplyr (Wickham et al. 2021)
- fastDummies (Kaplan 2020)
- ggcorrplot (Kassambara 2019)
- ggstance (Henry, Wickham, and Chang 2020)
- huxtable (Hugh-Jones 2021)
- jtools (Long 2020)
- lavaan (Rosseel 2012)
- semPlot (Epskamp 2019)
- tidySEM (van Lissa 2021)
- lme4 (Bates et al. 2015)
- performance (Lüdecke et al. 2021)

**References**

Douglas, B; Mächler, M; Bolker, B; & Walker, S. (2015). “Fitting Linear Mixed-Effects Models Using lme4.” Journal of Statistical Software *67* (1): 1–48. <https://doi.org/10.18637/jss.v067.i01>.

Epskamp, S.(2019). semPlot: Path Diagrams and Visual Analysis of Various SEM Packages’ Output. [https://CRAN.R-project.org/package=semPlot](https://cran.r-project.org/package=semPlot).

Fox, J; & Weisberg, S.(2019). An R Companion to Applied Regression. Third. Thousand Oaks CA: Sage. <https://socialsciences.mcmaster.ca/jfox/Books/Companion/>.

Gallucci, M. GMLj.(2020). General Analysis for Linear Models. Available online: <https://gamlj.github.io> (accessed on 11 November 2020)

Henry, L; Hadley, W: & Chang, W.(2020). Ggstance: Horizontal ’Ggplot2’ Components. <https://CRAN.R-project.org/package=ggstance>.

Hugh-Jones, D.(2021). Huxtable: Easily Create and Style Tables for LaTeX, HTML and Other Formats. [https://CRAN.R-project.org/package=huxtable](https://cran.r-project.org/package=huxtable).

Kaplan, J.(2020). fastDummies: Fast Creation of Dummy (Binary) Columns and Rows from Categorical Variables. [https://CRAN.R-project.org/package=fastDummies](https://cran.r-project.org/package=fastDummies).

Kassambara, A.(2019). Ggcorrplot: Visualization of a Correlation Matrix Using ’Ggplot2’. [https://CRAN.R-project.org/package=ggcorrplot](https://cran.r-project.org/package=ggcorrplot).

Long, J.(2020). Jtools: Analysis and Presentation of Social Scientific Data. <https://cran.r-project.org/package=jtools>.

Love, J.(2021). Jmvcore: Dependencies for the ’Jamovi’ Framework. [https://CRAN.R-project.org/package=jmvcore](https://cran.r-project.org/package=jmvcore).

Lüdecke, D.(2018). “Sjmisc: Data and Variable Transformation Functions.” Journal of Open Source Software 3 (26): 754. <https://doi.org/10.21105/joss.00754>.

Lüdecke, D.(2021a). Sjlabelled: Labelled Data Utility Functions (Version 1.1.8). <https://doi.org/10.5281/zenodo.1249215>.

Lüdecke, D: Mattan, S; Shachar, B; Patil, I; Waggoner, P; & Makowski, D.(2021). “performance: An R Package for Assessment, Comparison and Testing of Statistical Models.” Journal of Open Source Software 6 (60): 3139. <https://doi.org/10.21105/joss.03139>.

Makowski, D, Mattan S. Shachar, B; Patil, I; & Lüdecke, D.(2021). “Automated Results Reporting as a Practical Tool to Improve Reproducibility and Methodological Best Practices Adoption.” CRAN. <https://github.com/easystats/report>.

R Core Team.(2021). R: A Language and Environment for Statistical Computing. Vienna, Austria: R Foundation for Statistical Computing. [https://www.R-project.org/](https://www.r-project.org/).

Rodríguez-Sánchez, F; & Hutchins, S.D.(2020). Grateful: Facilitate Citation of r Packages. <https://github.com/Pakillo/grateful>.

Rosseel, Y.(2012). “lavaan: An R Package for Structural Equation Modeling.” Journal of Statistical Software 48 (2): 1–36. <https://www.jstatsoft.org/v48/i02/>.

Selker, R; Jonathon, L; Dropmann, D; & Moreno, V.(2021). Jmv: The ’Jamovi’ Analyses. [https://CRAN.R-project.org/package=jmv](https://cran.r-project.org/package=jmv).

Wei, T; & Simko, V.(2021). R package 'corrplot': Visualization of a Correlation Matrix (Version 0.90). Available from <https://github.com/taiyun/corrplot>

van Lissa, C.J.(2021). tidySEM: Tidy Structural Equation Modeling. [https://CRAN.R-project.org/package=tidySEM](https://cran.r-project.org/package=tidySEM).

Wickham, H.(2016). Ggplot2: Elegant Graphics for Data Analysis. Springer-Verlag New York. [https://ggplot2.tidyverse.org](https://ggplot2.tidyverse.org/).

Wickham, H; Averick, M; Bryan, J; Chang, W; McGowan, L.D; François, R; Grolemund, G .(2019). “Welcome to the tidyverse.” Journal of Open-Source Software 4 (43): 1686. <https://doi.org/10.21105/joss.01686>.

Wickham, H; & Bryan, J.(2019). Readxl: Read Excel Files. [https://CRAN.R-project.org/package=readxl](https://cran.r-project.org/package=readxl).

Wickham, H; François, R; Henry, L; & Müller, K.(2021). Dplyr: A Grammar of Data Manipulation. [https://CRAN.R-project.org/package=dplyr](https://cran.r-project.org/package=dplyr).
